# Uptake and translocation of pharmaceutically active compounds by olive tree (*Olea europaea* L.) irrigated with treated municipal wastewater

**DOI:** 10.3389/fpls.2024.1382595

**Published:** 2024-05-02

**Authors:** Alba N. Mininni, Angela Pietrafesa, Maria Calabritto, Roberto Di Biase, Gennaro Brunetti, Francesco De Mastro, Sapia Murgolo, Cristina De Ceglie, Carlo Salerno, Bartolomeo Dichio

**Affiliations:** ^1^ Department of European and Mediterranean Cultures, Environment, and Cultural Heritage (DICEM), University of Basilicata, Matera, Italy; ^2^ Department of Soil, Plant, and Food Science, University of Bari, Bari, Italy; ^3^ Department of Bari, Istituto di Ricerca Sulle Acque, CNR, Bari, Italy

**Keywords:** wastewater irrigation, water reuse, pharmaceuticals, carbamazepine, fluconazole, antibiotics, soil contamination, roots uptake

## Abstract

**Introduction:**

The use of treated municipal wastewater (TWW) represents a relevant opportunity for irrigation of agricultural crops in semi-arid regions to counter the increasing water scarcity. Pharmaceutically active compounds (PhACs) are often detected in treated wastewater, posing a risk to humans and the environment. PhACs can accumulate in soils and translocate into different plant tissues, reaching, in some cases, edible organs and entering the food chain.

**Methods:**

This study evaluated the uptake and translocation processes of 10 PhACs by olive trees irrigated with TWW, investigating their accumulation in different plant organs. The experiment was conducted in southern Italy, in 2-year-old plants irrigated with three different types of water: freshwater (FW), TWW spiked with 10 PhACs at a concentration of 200 µg L^−1^ (1× TWW), and at a triple dose (3× TWW), from July to October 2021. The concentration of PhACs in soil and plant organs was assessed, collecting samples of root, stem, shoot, leaf, fruit, and kernel at 0 (T0), 50 (T1), and 107 (T2) days of irrigation. PhACs extraction from soil and plant organs was carried out using the QuEChERS method, and their concentrations were determined by high-resolution mass spectrometry coupled with liquid chromatography.

**Results:**

Results of uptake factors (UF) showed a different behavior between compounds according to their physicochemical properties, highlighting PhACs accumulation and translocation in different plant organs (also edible part) in 1× TWW and 3× TWW compared to FW. Two PhACs, carbamazepine and fluconazole, showed interactions with the soil–plant system, translocating also in the aerial part of the plant, with a translocation factor (TF) greater than 1, which indicates high root-to-leaf translocation.

**Discussion:**

Findings highlight that only few PhACs among the selected compounds can be uptaken by woody plants and accumulated in edible parts at low concentration. No effects of PhACs exposure on plant growth have been detected. Despite the attention to be paid to the few compounds that translocate into edible organs, these results are promising for adapting wastewater irrigation in crops. Increasing knowledge about PhACs behavior in woody plants can be important for developing optimized wastewater irrigation and soil management strategies to reduce PhACs accumulation and translocation in plants.

## Graphical Abstract

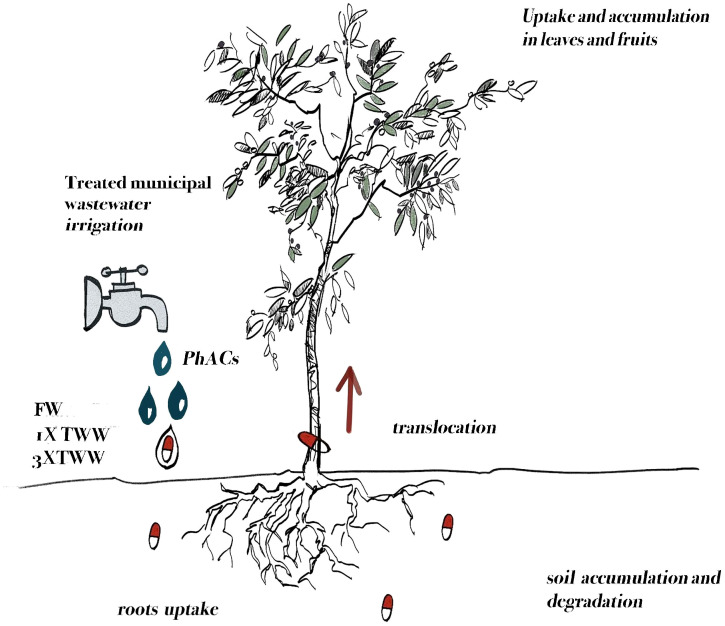

## 1 Introduction

Water is an important resource for human activities, i.e., agriculture, industry, and domestic use. According to the Food and Agriculture Organization (FAO) of the United Nations (UN), agriculture is the largest consumer of freshwater at the global level (approximately 70%), followed by industry (19%) and households (12%) (FAO, 2021). Irrigation land demand is expected to increase by 15% by 2030 (Hashem and Qi, 2021). On the other hand, freshwater scarcity will increase in the next few years and is one of the most important limiting factors for both crop production and food security. The increased warming trend and precipitation decline in the Mediterranean area make it a climate change hotspot (Cos et al., 2022), exacerbating water scarcity in agriculture (Chen et al., 2021; Al-Hazmi et al., 2023; Mishra et al., 2023; Oueslati et al., 2023). Alternative sources of water for crop irrigation rely on the use of unconventional sources, such as domestic and municipal wastewater. Wastewater reuse in crop irrigation is often practiced in arid and semi-arid countries, where evapotranspiration exceeds rainfall for most of the year (Bedbabis et al., 2014; Manasfi et al., 2021; Pérez et al., 2022), reaching 5%–12% of the total amount of treated effluent for crop irrigation (Shi et al., 2022). Municipal wastewater reuse for crop irrigation is estimated to be more than double in 2025 compared to 2000 in Europe (Lavrnić et al., 2017). Many studies consider the use of wastewater as a resource not only for crop irrigation, but also for fertilization, given its high nutrient content (Palese et al., 2006; Gargouri et al., 2022; Brunetti et al., 2023).

In fact, wastewater reuse has several beneficial effects, such as reducing the application and cost of chemical fertilizers, conserving freshwater resources, improving crop yields, and reducing environmental impact (Palese et al., 2009; Ungureanu et al., 2020; De Mastro et al., 2022; Lyu et al., 2022; Mininni et al., 2023). Although the benefits of wastewater use in agriculture are many, there are also several risks to humans and the environment related to the possible entry of contaminants and pollutants into the food chain. In particular, irrigation with wastewater could have negative impacts on soil properties and fertility (e.g., salinity, structural degradation, and reduced aeration), agricultural crops (accumulation of heavy metals and pathogens), and thus risks to human health (Bedbabis et al., 2014).

The use of wastewater in agriculture introduces numerous organic contaminants into the agroecosystem (e.g., in effluents and many surface waters). In recent years, the presence of a multitude of contaminants, known as contaminants of emerging concern (CECs), has become a global issue of growing environmental concern (Shi et al., 2022). CECs are mostly unregulated anthropogenic chemicals found in trace concentrations ranging from parts per trillion (ppt or ng L^−1^) to parts per billion (ppb or μg L^−1^) (Ben Mordechay et al., 2018; González García et al., 2019; Rout et al., 2021; Denora et al., 2023).

CECs can refer to various substances, including pharmaceutically active compounds (PhACs) that are widely used as analgesics, anesthetics, antibiotics, antimicrobials, anti-inflammatories, and antilipemics in human and veterinary fields; personal care products (perfumes, detergents, deodorants, and cleaning products); and flame retardants and pesticides (González García et al., 2018; Beltrán et al., 2020; Ferreira et al., 2020; Narain-Ford et al., 2022; Sunyer-Caldú et al., 2023). PhACs can be hazardous even at very low concentrations because they can enter the food chain and persist in the environment for a long time. The use of treated wastewater (TWW) in agriculture requires efficient wastewater treatment processes to improve the quality of treated water (Al-Hazmi et al., 2023), since PhACs are not completely removed by conventional processes adopted in wastewater treatment plants (WWTPs) (Ferreira et al., 2020).

Soil properties influence the adsorption process of PhACs, particularly the texture, soil organic matter (SOM) content, pH of the soil aqueous solution, and clay content and type, such as high percentages of clay minerals that enhance the adsorption of PhACs on the soil or uptake by plants (Wu et al., 2020).

Once in the soil, PhACs can undergo several processes that determine their fate: uptake-desorption, transformation, absorption, and translocation from soil to plant systems, accumulating in different hypogeal and/or aerial plant organs (Ben Mordechay et al., 2018; Chuang et al., 2019; Sunyer-Caldú et al., 2023). Potential uptake of PhACs by plants is affected by their mobility in soil and low sorption capacity, since PhACs in the soil solution represents the dominant fraction bioavailable to root uptake (Di Bonito et al., 2018; Li et al., 2022), following a pathway similar to that of mineral element uptake (Al-Farsi et al., 2017). Therefore, PhACs plant uptake and translocation are affected by several factors, such as their physicochemical properties (Pullagurala et al., 2018), including water solubility and octanol–water partition coefficient (log*K*_ow_), bioavailability in the media, exposure condition and time, and plant intrinsic properties and their metabolism (Carter et al., 2014; Al-Farsi et al., 2017). Differences in plant uptake were also related to peculiar plant physiology, determining different accumulation, uptake, and translocation mechanisms among vegetables (Goldstein et al., 2014). PhACs can be translocated through the root cells following three different pathways: via apoplastic, along cell walls through the intercellular space; via symplastic, between cells through interconnecting plasmodesmata; and transmembrane, between cells through cell walls and membranes (Miller et al., 2016). Once absorbed, PhACs can be transported to shoots, leaves, and fruits through the xylem or phloem, and subsequently enter the food chain, thus posing a potential health risk to humans and the livestock that feed on them (Bueno et al., 2022; Mininni et al., 2023). The distribution of PhACs in the irrigation water–soil–plant system is generally assessed through the determination of the uptake factors (UF), which are commonly referred to as the ratio of PhACs concentration in crop to that in soil and in water, considered as soil pore water (Carter et al., 2014; Li et al., 2019) and irrigation water (Qassim et al., 2020; Camacho-Arévalo et al., 2021; Manasfi et al., 2021; Bueno et al., 2022; Sunyer-Caldú et al., 2022). In particular, PhACs movement within the plant is evaluated through the translocation factor (TF), defined as the ratio of PhACs concentration in leaves to that in roots (Li et al., 2019; Beltrán et al., 2020; Camacho-Arévalo et al., 2021). In addition, PhACs are potentially toxic to plants, as they exert effects on plant morphological and physiological responses (Hurtado et al., 2017), affecting the development and overall physiology, depending on the type and properties of PhACs and exposure concentration (Christou et al., 2019a). Carvalho et al. (2014) reported the effects of the exposure to high concentrations of carbamazepine (>10 mg L^−1^) in cucumber plants, showing a reduction in total plant biomass, leaf size, and primary root length, and a change in secondary root shape and number. The development of wastewater irrigation management strategies and the use of efficient wastewater treatment processes to limit the accumulation of PhACs in soil and plant tissues are important to achieve safe food production and environment (Chen et al., 2021).

There are numerous experimental studies focused on the use of wastewaters for irrigation of various agricultural crops and the evaluation of PhACs uptake by plants. These studies particularly concerned the annual herbaceous crops, including artichoke, lettuce, tomatoes, carrots, cabbage, and others such as corn and wheat (Riemenschneider et al., 2017; Hammad et al., 2018; Chuang et al., 2019; Christou et al., 2019a; Pérez et al., 2022; Denora et al., 2023; Sunyer-Caldú et al., 2023). In contrast, little is still known about the uptake and translocation of PhACs in woody perennials and about the fate and effects of contaminants in general (soil and plant) (Narain-Ford et al., 2022). A lower concentration of carbamazepine was observed in citrus leaves than in leaves of leafy vegetables or tomatoes, probably due to the different plant physiology and processes that differ between trees and annual plants (Ben Mordechay et al., 2022). The uptake and distribution of PhACs in the soil–plant system of woody plants irrigated with TWW have not yet been thoroughly investigated. According to previous estimates based on the analysis of previous studies, the uptake and accumulation of PhACs by crop plants decrease in the order of leaf vegetables > root vegetables > cereal and fodder crops > fruit vegetables (Christou et al., 2019b).

This work intended to increase knowledge about the behavior of 10 PhACs in the wastewater–soil–plant system in woody perennials (olive tree) irrigated with treated municipal wastewater. The main aim was to determine the PhACs uptake and translocation dynamics and their accumulation from wastewater to soil and plant.

## 2 Materials and methods

### 2.1 Plant material and experimental design

The olive tree (*Olea europaea* L. cv. Frantoio) was chosen to study PhACs translocation and accumulation in plant organs following root uptake. Sixty 2-year-old plants were grown in 18-L pots containing sandy loam soil, during 2021 (from May to the end of October). Soil properties are reported in **Table 1**.

**Table 1:** Physicochemical properties of the soil substrate contained in pots.

Parameter	Value
Soil skeleton	Not present
Sand (%)	74
Silt (%)	12
Clay (%)	14
Textural class	Sandy loam
Soil organic carbon (%)	5.0
CaCO_3_ total content (%)	5.1
CaCO_3_ active (%)	2.4
pH (1:2.5)	7.7
Electrical conductivity at 25°C (1:2:0) (mS cm^−1^)	3.9
Total nitrogen (N) %	0.4
Available phosphorus (P) mg kg^−1^	141
Exchangeable potassium (K) mg kg^−1^	586

The experimental study was conducted in 2021 at the ALSIA Research Institute located in the Metapontino area (N 40° 23′, E 16° 47′) (Basilicata region, south Italy). During the 2021 season, the average temperature ranged from 2 to 15°C and 20 to 31°C in winter and summer, respectively, with high environmental evaporative demand during the hottest months and rainfall occurring mainly from November to April, reflecting a typically Mediterranean climate characterized by moderate, wet winters and hot, dry summers (**Supplementary Figure S1**).

**Figure 1:**
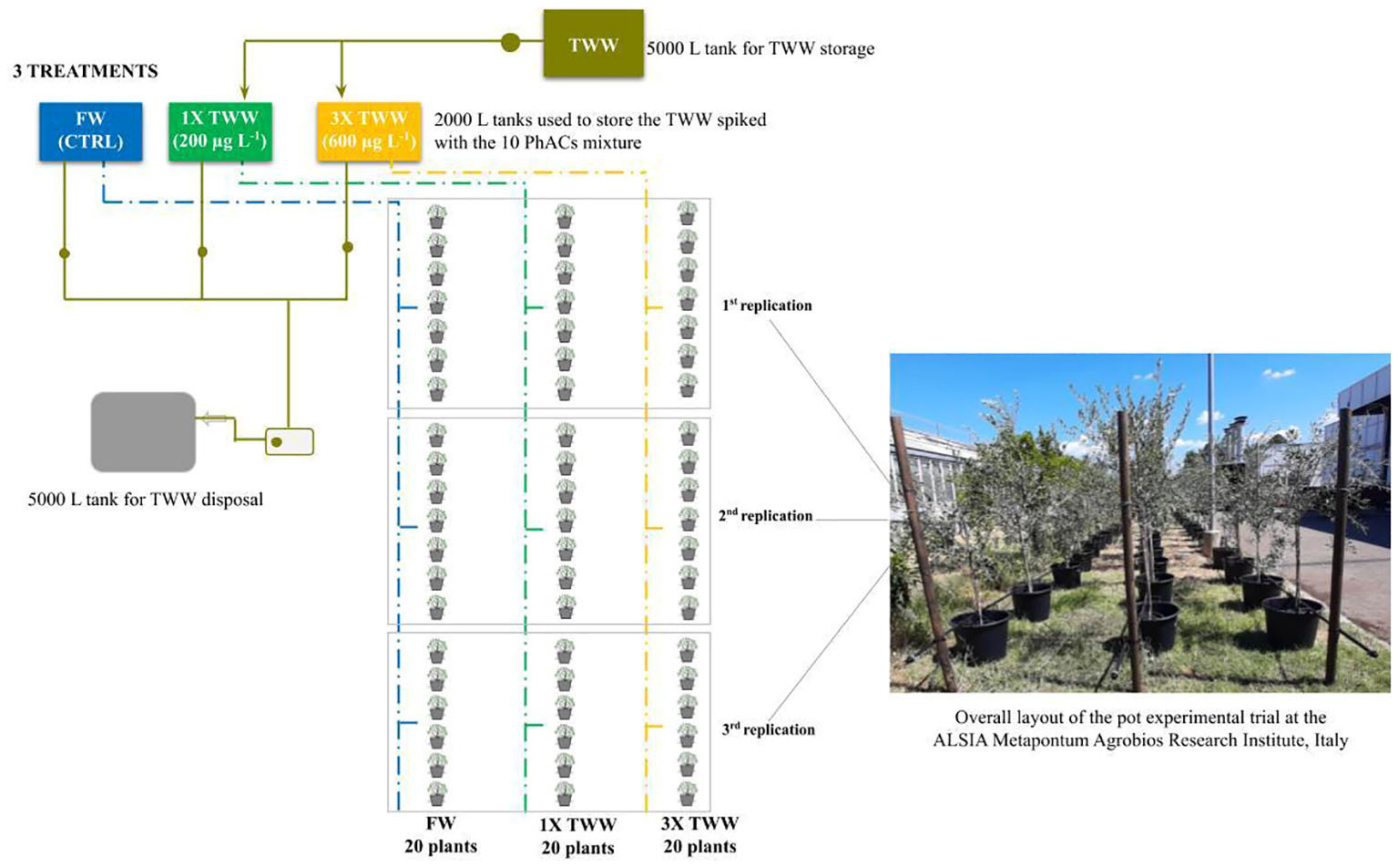
Experimental setup of the pot trial: tanks used for the storage of freshwater (FW) (blue) and the treated wastewater without PhACs (TWW, olive green), 1× TWW (green), and 3× TWW (orange) and tanks used for the safe disposal (light gray).

Twenty plants of uniform size were selected for each treatment [freshwater (FW); treated municipal wastewater spiked with 200 µg L^−1^ of each selected PhAC, 1× TWW, in a dose higher than the global average concentration in TWW (Golovko et al., 2021) to detect the PhACs; and treated municipal wastewater spiked with 600 µg L^−1^ of each selected PhAC, 3× TWW, simulating extreme conditions to investigate the uptake and translocation of PhACs in plant] and distributed according to the experimental design (**Figure 1**) to compare the three irrigation treatments. Acclimation period lasted 2 months (May and June 2021), during which all plants were irrigated with FW. Irrigation with TWW treatments started on 1 July 2021.TWW was the final effluent produced by a disinfection step through peracetic acid (2.5 mg L^−1^) of a secondary effluent arrived from a municipal WWTP located at Ferrandina (Basilicata region, Italy, Palese et al., 2009; Sofo et al., 2019), transported every 15 days from Ferrandina to Metaponto, then stocked in a 5,000-L tank (Ø 224 cm and H 140 cm).

TWW was stored for a maximum of 2 days before being transferred in the 2,000-L tanks (Ø 170 cm and H 120 cm) for 1× and 3× TWW treatments where the mix solution of the 10 selected PhACs was manually added to TWW, according to defined concentrations, every 15 days during the irrigation season. The tanks were made of dark green polyethylene (PE) for protection from sunlight and were resistant to temperature changes between −40°C and +60°C (**Figure 1**).

#### 2.1.1 Irrigation regime

The surface drip-irrigation system with a PE irrigation pipe was chosen for this experiment, making roots the only efficient PhACs uptake pathway. The plants were drip-irrigated with four emitters per pot to ensure a uniform soil wetting. Each pot was put inside a closed vessel to collect the percolation water and add it back to the pot, ensuring a closed irrigation system. The cumulative irrigation volume was approximately 150 L plant^−1^ (p^−1^) for all irrigation treatments, defined according to plant water needs, providing daily irrigation volumes ranging from 2.5 L p^−1^ to 1.0 L p^−1^ in August and October, respectively, to compensate for evapotranspiration losses and ensure a soil water content close to field capacity. The daily irrigation volume was divided into two/five events (each of 0.5 L p^−1^) depending on the environmental evaporative demand. The irrigation was optimized to avoid the occurrence of waterlogging or water deficit. The drip irrigation system complies with the guidelines for a safe reuse of TWW for irrigation and is considered one of the most efficient and common irrigation systems adopted, especially in arid and semi-arid regions (Manasfi et al., 2021).

#### 2.1.2 Chemicals

Ten PhACs commonly detected in wastewater at concentrations ranging from a few ng L^−1^ to a few µg L^−1^ (Chen et al., 2013; Rogowska et al., 2020; Ben Mordechay et al., 2021; Sunyer-Caldú et al., 2023) and belonging to five classes, namely, antibiotics (clarithromycin, sulfamethoxazole, and trimethoprim), antiepileptic (carbamazepine), anti-inflammatory (ketoprofen, diclofenac, and naproxen), antifungal (fluconazole and climbazole), and beta-blocker (metoprolol), were selected. The selection was based on the occurrence of these compounds in municipal wastewaters and their inefficient removal during conventional treatments (Ben Mordechay et al., 2022). The physicochemical characteristics and chemical structures of the selected PhACs are reported in **Supplementary Table S1**. The standards (>98% purity) were used to prepare all the 10 compound stock standard solutions (1,000 ppm) in methanol. The standard solution was added to wastewater in the tanks to achieve two concentrations: 200 μg L^−1^ (1× TWW) and 600 μg L^−1^ (3× TWW) of each compound.

### 2.2 Irrigation water analysis

During the experimental period, FW and TWW were sampled once per month directly from the corresponding tanks and analyzed in terms of conventional physicochemical properties (**Table 2**).

**Table 2:** Average values with standard deviation of the conventional physicochemical parameters of the used FW and TWW (*n* = 4).

Parameter	Unit	FW	TWW
Total suspended solids (TSS)	mg L^−1^	21.5 ± 20.7	13.4 ± 9.6
Turbidity	NTU	22.1 ± 23.6	8.9 ± 5.4
Chemical oxygen demand (COD)	mg O_2_ L^−1^	12.3 ± 4.9	38.0 ± 23.8
Biochemical oxygen demand at day 5 (BOD_5_)	mg O_2_ L^−1^	3.9 ± 0.6	15.8 ± 12.4
Nitrate	mg N L^−1^	1.2 ± 0.9	5.8 ± 4.4
Total phosphorus	mg P L^−1^	0.1 ± 0.1	2.6 ± 1.4
pH	–	8.0 ± 0.3	7.5 ± 0.3
Electrical conductivity	mS cm^−1^	0.9 ± 0.0	0.9 ± 0.1

### 2.3 PhACs analytical determination in soil and plant organs

Soil and plant organ samples were collected immediately before the application of 1× TWW and 3× TWW irrigation treatments on 30 June 2021 (T0, early fruit development), again after 50 days (T1, fruit development—pit hardening), and at harvest in October, after 107 days (T2, fruit maturity) from the application of irrigation treatments. Three replicates of soil per treatment were collected using an auger sampler bulking eight different soil sampling points at a depth between 0 and 25 cm. Each composite sample was air-dried at room temperature, sieved at 2 mm, and stored in 50-mL sterilized plastic containers in the dark at −20°C until analysis.

Plant organs were sampled by choosing one plant from each replication group (first, second, and third, *n* = 3) per treatment, according to uniform size and plant status. The following plant organs were collected: fine roots (1–5 mm in diameter) and 1-year vegetative shoots, leaves, stem, and olive fruits from which pulp and kernel were separated. Three plants per treatment were destroyed at T0 and T2 for sample collection and biomass partitioning assessment. Each organ from the aerial and hypogeal parts was separated for fresh biomass and dry matter measurements. Roots were thoroughly rinsed with ultrapure deionized water to remove any adhered particles and subsequently dried with paper. The plant organs were finely chopped with a household blender at high speed to obtain a fine-grained and homogenized material that was stored in 50-mL sterilized plastic containers at −20°C until analysis. The extraction of PhACs from soils and plant organs was performed with the QuEChERS analytical method (De Mastro et al., 2022; Brunetti et al., 2023; De Mastro et al., 2023). The QuEChERS method is more advantageous than traditional extraction methods in terms of extraction time and equipment required. Briefly, 2 g of fresh sample (olive roots, leaves, shoots, stems, pulp, and kernel) was placed in a centrifuge tube. MilliQ water (6 mL) was added to the centrifuge tube followed by capping and shaking vigorously for 1 min. After the sample was thoroughly wetted, 10 mL of acetonitrile was added to the centrifuge tube and shaken by hand for 5 min. Then, a salting-out with citrate buffer (4 g MgSO_4_, 1 g NaCl, 0.5 g NaCitrate dibasic sesquihydrate, and 1 g NaCitrate tribasic dihydrate) was performed. After the addition of the salt, the tube was immediately manually shaken for 5 min and subsequently centrifuged (5 min, 3,700 rpm), allowing a phase separation between the aqueous and organic solvents. After that, 6 mL of the upper acetonitrile layer was transferred into a new 15-mL tube for the clean-up step. Tubes containing 900 mg of MgSO_4 + _150 mg of PSA (primary secondary amine) for root samples and 900 mg of MgSO_4_ + 150 mg of PSA + 150 mg of octadecyl (C18) for shoots, leaves, stems, pulp, and kernel were vortexed for 1 min and subsequently centrifuged (5 min, 3,700 rpm). The supernatant (1.5 mL) was filtered with a membrane filter (PVDF, 0.22 μm) and was transferred into a screw cap vial for analysis. The analytical technique of high-resolution mass spectrometry coupled with liquid chromatography (LC-HRMS/MS) was used to determine the concentration of PhACs for the three replicates of each treatment. In detail, an Ultimate 3000 System (Thermo Fisher Scientific) was interfaced to a high-resolution mass spectrometer, TripleTOF 5600+ system (AB Sciex) equipped with a duo-spray ion source operated in positive electrospray ionization (ESI) mode. The MS parameters were set as follows (arbitrary units): nebulizer gas 35, turbo gas 45, curtain gas 20, ion spray voltage 5,500 V, temperature 500°C, declustering potential 80 V, collision energy voltage 35 V, collision energy spread voltage 15 V, and *m*/z range 70–800 Da. An acquisition method based on TOF-MS/IDA (Information Dependent Acquisition) experiments, in the mass scan range of 70–800 Da, was optimized for the detection of analytes.

As far as the soil and olive tree extracts, 100 µL of each sample was injected into the LC-MS system employing a ZORBAX Eclipse Plus C18 column (150 × 2.1 mm, 1.8 µm) operating at a flow rate of 0.300 mL min^−1^, and the chromatographic separation of analytes was reached employing the mobile phases 0.1% formic acid in water (solvent A) and 0.1% formic acid in acetonitrile (solvent B) with the following gradient: 0−2 min, 2% solvent B; 2−3 min, linear from 2% to 20% solvent B; 3−17 min, linear from 20% to 100% solvent B; 17−21 min, isocratic at 100% solvent B; 21−21.5 min, from 100% to 2% solvent B; 21.5−25 min, column reconditioning. Before analysis, both extracts and standards were spiked with an internal standard, i.e., Carbamazepine D10 at a level of 10 µg L^−1^, to evaluate the relative reduction in the signal intensity due to the matrix effect. The linearity of the analytical method was validated in the range 0.1–10 μg L^−1^ (0.1, 0.2, 0.5, 1, 2, 5, and 10 μg L^−1^) obtaining determination coefficient values ≥ 0.98.

As for water samples, the chromatographic method was implemented with an initial online solid-phase extraction (SPE) method by a 10-port rheodyne valve, 2 positions (1-2 and 1-10) based on the injection of 2,000 μL of each sample, previously filtered with a 0.20-μm regenerated cellulose filter, through a Hypersil GOLDaQ column (20 × 2.1 mm, 5 μm) operating at a flow rate of 0.250 mL min^−1^ using 100% water to obtain the enrichment of the analytes. The subsequent chromatographic separation of analytes was achieved by employing the LC condition described above. The SPE online method allowed an increase in the sensitivity of the analytical method for the detection of the target PhACs detected in traces in the investigated treated municipal wastewater. Before analysis, both water samples and standards were spiked with an internal standard, i.e., Carbamazepine D10 at a level of 1 µg L^−1^, and the linearity of the analytical method was validated in the range 0.01–10 μg L^−1^ (0.01, 0.05, 0.1, 0.5, 1, 2, 5, and 10 μg L^−1^) obtaining determination coefficient values ≥ 0.98.

The limit of quantification (LOQ) for each target PhAC (**Table 3**) was determined based on the lowest calibration standard with a signal-to-noise ratio of the qualifier ion higher than 10:1.

**Table 3:** Limit of quantification (LOQ) of pharmaceutically active compounds in waters (μg L^−1^), soil, and olive tree extracts (ng g^−1^).

PhACs	Chemical formula	[M+H]^+^ (m/z)	Retention time (min)	Source employed/polarity	LOQ		
					Waters (µg L^−1^)	Soil (ng g^−1^)	Olive tree extracts (ng g^−1^)
Clarithromycin	C_38_H_69_NO_13_	748.4842	7.1	ESI (+)	0.01	0.5	0.8
Sulfamethoxazole	C_10_H_11_N_3_O_3_S	254.0594	7.1	ESI (+)	0.05	0.5	0.8
Trimethoprim	C_14_H_18_N_4_O_3_	291.1452	4.4	ESI (+)	0.01	0.5	0.8
Ketoprofen	C_16_H_14_O_3_	255.1016	10.3	ESI (+)	0.01	0.5	0.8
Carbamazepine	C_15_H_12_N_2_O	237.1022	8.4	ESI (+)	0.01	0.5	0.8
Diclofenac	C_14_H_11_Cl_2_NO_2_	296.0240	12.2	ESI (+)	0.05	3.0	4.0
Metoprolol	C_15_H_25_NO_3_	268.1907	4.7	ESI (+)	0.01	0.5	0.8
Fluconazole	C_13_H_12_F_2_N_6_O	307.1113	5.9	ESI (+)	0.01	0.5	0.8
Climbazole	C_15_H_17_ClN_2_O_2_	293.1051	7.3	ESI (+)	0.01	0.5	0.8
Naproxen	C_14_H_14_O_3_	231.1016	10.4	ESI (+)	0.10	3.0	4.0

### 2.4 Irrigation water–soil–plant system: PhACs uptake and translocation factors

In order to estimate the ability of olive plants to uptake and accumulate PhACs from soil irrigated with spiked TWW (1× and 3× TWW), UF related to soil were calculated for each PhAC. UF, derived from PhACs concentrations detected in the soil–plant system, were calculated at T1 and T2 according to Equation 1 (Qassim et al., 2020; Camacho-Arévalo et al., 2021; Manasfi et al., 2021; Bueno et al., 2022; Sunyer-Caldú et al., 2022):


(1) $$UF=\frac{Cplant}{Csoil}$$

where UF is the soil-based uptake factor, *C*_plant_ is the contaminant concentration in the different plant organs (roots, leaves, shoots, stem, pulp, and kernel), and *C*_soil_ is the contaminant concentration in the soil.

The soil–water sorption coefficient for each PhAC was obtained considering the average of *C*_soil_ at T1 and T2 for 1× TWW and 3× TWW according to the Equation 2 (Sunyer-Caldú et al., 2022):


(2) $$Kd=\frac{Csoil}{Cwater}$$

where *C*_water_ is the contaminant concentration of the spiked irrigation wastewater, 200 and 600 μg L^−1^ for 1× and 3× TWW, respectively.

The TF, which indicates the translocation of the PhACs from the roots to the leaves, was calculated as the ratio between the concentration of PhACs in the aerial organs of the plant (*C*_leaf_) and their respective concentrations in roots (*C*_root_), according to Li et al. (2019); Beltrán et al. (2020), and Camacho-Arévalo et al. (2021). The TF was calculated according to the Equation 3:


(3) $$TF=\frac{Cleaf}{Croot}$$

### 2.5 Plant growth

The effects of PhACs on plant growth were assessed by measuring plant organ biomass partitioning, expressed as fresh weight (g) and dry matter (%). At the end of the experimental trial, in October 2021, three plants per irrigation treatment (FW, 1× TWW, and 3× TWW) were destroyed to measure plant organ biomass.

### 2.6 Data and statistical analysis

One-way analysis of variance (ANOVA) was used to examine the differences between PhACs concentration at each sampling time, after statistically verifying the normality of distribution (Shapiro–Wilk normality test). Data that were not normally distributed were log transformed for normalization. Differences among means were identified by Tukey’s pairwise comparison test and *p*-value<0.05 was considered significant. Statistical analyses were conducted using the RStudio statistical software (4.1.3 version, Posit Software, PBC, Boston, MA) and results were plotted with SigmaPlot 15.0 (Systat Software Inc., San Jose, CA, USA). Data were reported as mean value and standard error of the mean (± SE).

## 3 Results

### 3.1 PhACs concentrations in soil and plant organs over time

**Figure 2:**
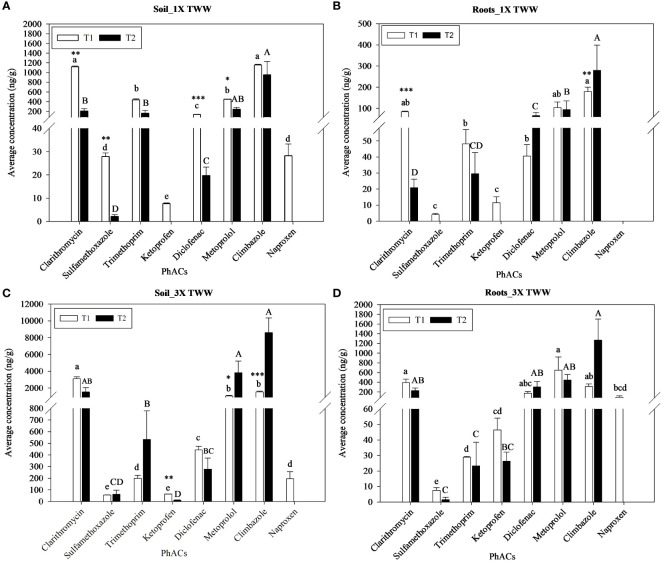
Average concentrations (ng g^−1^) of the PhACs detected in soil and roots irrigated with 1× TWW **(A, B)** and 3× TWW **(C, D)** treatments at sampling times T1 and T2. Data are reported as mean values and bars are standard error of the mean (*n* = 3). Different lowercase letters indicate significant differences between PhACs concentration at the exposure time T1. Different capital letters indicate significant differences between PhACs concentration at the exposure time T2. Asterisks denote significant differences between exposure times within the same PhAC according to a one-way ANOVA and Tukey with *p*< 0.05. Significant codes: *p*< 0.05 (*), *p*< 0.01 (**), and *p*< 0.001 (***).

No PhACs were detected in all samples collected per irrigation treatment at T0. In soil and plant organs of non-exposed control plants, i.e., irrigated with FW, the PhACs detected over time were below the LOQ. The average concentrations of each analyzed PhAC in soil and roots irrigated with 1× TWW and 3× TWW treatments at T1 and T2 are presented in **Figure 2**.

Otherwise, most PhACs were detected at concentrations above their LOQs in soil samples irrigated with 1× TWW and 3× TWW irrigation treatments at both T1 and T2 (**Figures 2A, C**). Two PhACs, namely, ketoprofen and naproxen, in 1× TWW and only naproxen in 3× TWW were found in the soil at T1 but not at T2 (**Figures 2A, C**). Clarithromycin and climbazole were found at similar concentrations in soil, significantly higher compared to other compounds, followed by trimethoprim and metoprolol, diclofenac, sulfamethoxazole, naproxen, and ketoprofen in the soil samples collected from plants irrigated with 1× TWW at T1 (**Figure 2A**). Similarly, clarithromycin was found at the highest concentration, followed by metoprolol and climbazole, diclofenac, naproxen and trimethoprim, sulfamethoxazole, and ketoprofen in the soil samples collected from plants irrigated with 3× TWW at T1 (**Figure 2C**). Most PhACs showed a significantly higher concentration in soils at T1 than at T2, such as clarithromycin, sulfamethoxazole, diclofenac, and metoprolol in the 1× TWW (**Figure 2A**). In contrast, two PhACs, namely, metoprolol and climbazole, showed a significantly higher concentration in T2 soils than in T1 soils for the 3× TWW irrigation treatment (**Figure 2C**).

Also for soil, almost all PhACs were likewise detected in roots at both T1 and T2 and for both irrigation treatments. As shown in **Figure 2B**, naproxen was the only compound not detected at both sampling times, while two PhACs, i.e., sulfamethoxazole and ketoprofen, were below the LOQ at T2 in the 1× TWW. Instead, only naproxen remained below the LOQ at T2 in the 3× TWW irrigation treatment (**Figure 2D**). Climbazole, metoprolol, and clarithromycin showed a concentration in roots higher than other compounds, while sulfamethoxazole and ketoprofen showed the lowest concentration at T1 in the 1× TWW irrigation treatment (**Figure 2B**). A similar sequence was observed at T1 in the 3× TWW (**Figure 2D**). Among the different sampling times, the clarithromycin concentration in roots significantly decreased at the end of the irrigation season (T2), while climbazole increased, showing the highest concentration compared to other compounds in the 1× TWW irrigation treatment (**Figure 2B**). Moreover, sulfamethoxazole, trimethoprim, ketoprofen, and metoprolol were higher at T1 than at T2, while diclofenac was higher at T2, although no significant differences were detected for these compounds. No significant differences were found among the different sampling times in the 3× TWW (**Figure 2D**), but the behavior of compounds reflected results obtained in 1× TWW.

PhACs levels in soil and roots increased significantly in the 3× TWW compared to the 1× TWW irrigation treatment, except for sulfamethoxazole and trimethoprim showing similar and even opposite concentrations, respectively, from the comparison of the two irrigation treatments (**Figure 2**).

Most PhACs did not translocate and accumulate in the aerial plant organs, since they were detected below the LOQ (<LOQ) in both the 1× TWW and 3× TWW treatments.

**Figure 3:**
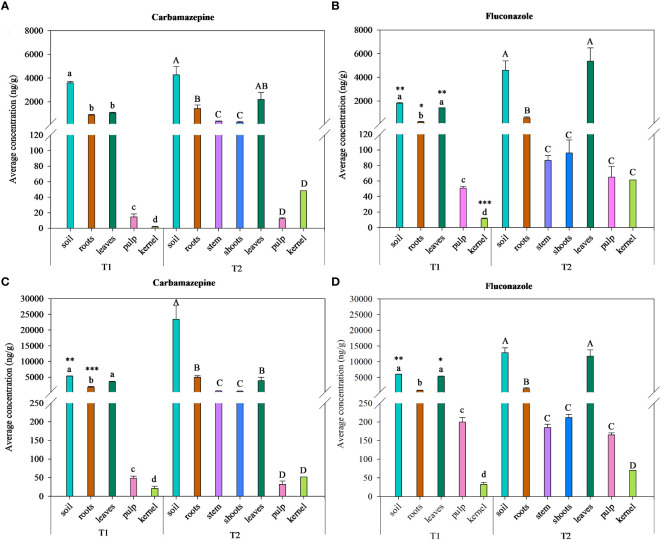
Average concentrations (ng g^−1^) for carbamazepine and fluconazole in the soil–plant system irrigated with 1× TWW **(A, B)** and 3× TWW **(C, D)** treatments at times T1 and T2. Data are reported as mean values and bars are standard errors of the mean (*n* = 3). Different lowercase letters indicate significant differences between soil and plant organs at the exposure time T1. Different capital letters indicate significant differences between soil and plant organs at the exposure time T2. Asterisks denote significant differences between exposure times within each soil and plant organs according to a one-way ANOVA and Tukey with *p*< 0.05. Significant codes: *p*< 0.05 (*), *p*< 0.01 (**), and *p*< 0.001 (***).

Two PhACs, i.e., carbamazepine and fluconazole, were found in the aerial plant organs (**Figure 3**). Values measured revealed a potential accumulation and translocation of these PhACs, distributed through 1× TWW and 3× TWW irrigation treatments, from soil to plant organs. Carbamazepine and fluconazole were the PhACs found at the highest concentrations in soil in both irrigation treatments, and their concentration increased over time (**Figure 3**).

The highest concentrations of carbamazepine and fluconazole were observed in roots and leaves among plant organs at both sampling times and irrigation treatments (**Figure 3**). No significant differences were observed between T1 and T2 in the carbamazepine concentration in soil and plant organs subjected to the 1× TWW irrigation treatment (**Figure 3A**), while concentrations in soil and roots subjected to the 3× TWW irrigation treatment significantly increased at T2 (**Figure 3C**). Significant differences were observed for fluconazole already in the 1× TWW irrigation treatment, where concentrations in soil, leaves, and roots significantly increased at T2 (**Figure 3B**). Similarly, concentrations in soil and leaves increased at T2 also in the 3× TWW irrigation treatment with the exception of root concentration, which maintained steady values between T1 and T2 (**Figure 3D**). Carbamazepine was found in the soil–plant system at T2 at concentrations as follows: soil > leaves > roots > stem > shoots > kernel > pulp in the 1× TWW irrigation treatment (**Figure 3A**) and soil > roots > leaves > stem > shoots > kernel > pulp in the 3× TWW irrigation treatment (**Figure 3C**). Fluconazole was found in the soil–plant system at T2 at concentrations as follows: leaves > soil > roots > shoots > stem > pulp > kernel in the 1× TWW irrigation treatment (**Figure 3B**) and soil > leaves > roots > shoots > stem > pulp > kernel in the 3× TWW irrigation treatment (**Figure 3D**). The results suggest that both compounds accumulated at higher concentrations in leaves and roots compared to other plant organs, and in particular, fluconazole had a greater capacity to move from roots to leaves compared to carbamazepine. Concentrations of carbamazepine and fluconazole in the soil–plant system were higher in the 3× TWW than in 1× TWW irrigation treatment.

The antiepileptic carbamazepine and the antifungal fluconazole were found in the edible parts of the plant, i.e., pulp and kernel, at both T1 and T2 and for both irrigation treatments. Concentrations of pulp samples of approximately 12–14 and 50–65 ng g^−1^ in the 1× TWW, and 30–50 and 165–200 ng g^−1^ in the 3× TWW, were measured for carbamazepine and fluconazole, respectively. No significant differences were found between sampling times in the increase or decrease in accumulation of these compounds within the edible part of the plant in both irrigation treatments.

### 3.2 Uptake and translocation factors

To better understand the potential uptake of PhACs by olive plants and their accumulation in the soil–plant system, UF values were calculated per PhAC, irrigation treatment, and sampling time considering the concentration in plant organs related to that in soil (UF) (**Table 4**).

**Table 4:** Soil-water sorption coefficients (*K*_d_) and uptake factors (UF) for each PhAC calculated in olive trees irrigated with 1× TWW and 3× TWW at T1 and T2.

PhACs	Treatment	*K*_d_ (mL/g)	T1				T2					
			UF (g/g)				UF (g/g)					
			Roots (1–5 mm)	Leaves	Pulp	Kernel	Roots (1–5 mm)	Stems	Shoots 1 year	Leaves	Pulp	Kernel
Clarithromycin	1× TWW	3.32 ± 1.03	0.08 ± 0.00	<LOQ	<LOQ	<LOQ	0.11 ± 0.03	<LOQ	<LOQ	<LOQ	<LOQ	<LOQ
	3× TWW	3.87 ± 0.71	0.13 ± 0.03	<LOQ	<LOQ	<LOQ	0.19 ± 0.08	0.003 ± 0.00	<LOQ	<LOQ	<LOQ	<LOQ
Sulfamethoxazole	1× TWW	0.08 ± 0.03	0.15 ± 0.03	<LOQ	<LOQ	<LOQ	<LOQ	<LOQ	<LOQ	<LOQ	<LOQ	<LOQ
	3× TWW	0.10 ± 0.03	0.13 ± 0.03	<LOQ	<LOQ	<LOQ	0.03 ± 0.00	<LOQ	<LOQ	<LOQ	<LOQ	<LOQ
Trimethoprim	1× TWW	1.50 ± 0.34	0.11 ± 0.02	<LOQ	<LOQ	<LOQ	0.37 ± 0.27	<LOQ	<LOQ	<LOQ	<LOQ	<LOQ
	3× TWW	0.61 ± 0.22	0.15 ± 0.01	<LOQ	<LOQ	<LOQ	0.05 ± 0.03	<LOQ	<LOQ	<LOQ	<LOQ	<LOQ
Ketoprofen	1× TWW	0.02 ± 0.01	1.53 ± 0.50	<LOQ	<LOQ	<LOQ	<LOQ	<LOQ	<LOQ	<LOQ	<LOQ	<LOQ
	3× TWW	0.06 ± 0.02	0.72 ± 0.12	<LOQ	<LOQ	<LOQ	2.52 ± 0.54	<LOQ	<LOQ	<LOQ	<LOQ	<LOQ
Carbamazepine	1× TWW	19.69 ± 1.76	0.25 ± 0.02	0.30 ± 0.00	0.004 ± 0.00	0.0005 ± 0.00	0.33 ± 0.04	0.09 ± 0.01	0.07 ± 0.02	0.52 ± 0.13	0.003 ± 0.00	0.01 ± 0.00
	3× TWW	24.03 ± 7.45	0.35 ± 0.02	0.66 ± 0.02	0.01 ± 0.00	0.004 ± 0.00	0.22 ± 0.03	0.02 ± 0.00	0.02 ± 0.00	0.17 ± 0.04	0.001 ± 0.00	0.002 ± 0.00
Diclofenac	1× TWW	0.39 ± 0.13	0.30 ± 0.05	<LOQ	<LOQ	<LOQ	3.63 ± 1.14	<LOQ	<LOQ	<LOQ	<LOQ	<LOQ
	3× TWW	0.60 ± 0.10	0.40 ± 0.09	<LOQ	<LOQ	<LOQ	1.24 ± 0.45	<LOQ	<LOQ	<LOQ	<LOQ	<LOQ
Metoprolol	1× TWW	1.74 ± 0.25	0.23 ± 0.06	<LOQ	<LOQ	<LOQ	0.45 ± 0.25	<LOQ	<LOQ	<LOQ	<LOQ	<LOQ
	3× TWW	4.07 ± 1.46	0.63 ± 0.07	0.02 ± 0.00	<LOQ	<LOQ	0.16 ± 0.08	<LOQ	<LOQ	0.0004 ± 0.00	<LOQ	<LOQ
Fluconazole	1× TWW	16.04 ± 3.59	0.13 ± 0.03	0.79 ± 0.03	0.03 ± 0.00	0.01 ± 0.00	0.14 ± 0.05	0.02 ± 0.01	0.02 ± 0.00	1.16 ± 0.13	0.02 ± 0.00	0.01 ± 0.00
	3× TWW	15.72 ± 2.81	0.15 ± 0.01	0.89 ± 0.02	0.03 ± 0.00	0.01 ± 0.00	0.12 ± 0.03	0.01 ± 0.00	0.02 ± 0.02	0.92 ± 0.12	0.01 ± 0.00	0.01 ± 0.00
Climbazole	1× TWW	5.27 ± 0.66	0.16 ± 0.02	<LOQ	<LOQ	<LOQ	0.33 ± 0.19	<LOQ	<LOQ	<LOQ	<LOQ	<LOQ
	3× TWW	8.46 ± 2.94	0.21 ± 0.04	<LOQ	<LOQ	<LOQ	0.15 ± 0.06	<LOQ	<LOQ	<LOQ	<LOQ	<LOQ
Naproxen	1× TWW	0.08 ± 0.03	<LOQ	<LOQ	<LOQ	<LOQ	<LOQ	<LOQ	<LOQ	<LOQ	<LOQ	<LOQ
	3× TWW	0.17 ± 0.08	0.55 ± 0.27	<LOQ	<LOQ	<LOQ	<LOQ	<LOQ	<LOQ	<LOQ	<LOQ	<LOQ

Data are expressed as the mean value (*n* = 3 ± SE).

The average soil–water sorption coefficient (*K*_d_) values ranged from 0.02 to 19.69 mL g^−1^ in the 1× TWW, and from 0.06 to 24.03 mL g^−1^ in the 3× TWW. *K*_d_ values measured in 1× TWW were in the following order: ketoprofen< sulfamethoxazole, naproxen< diclofenac< trimethoprim, metoprolol< clarithromycin< climbazole< fluconazole, carbamazepine, while in 3× TWW: ketoprofen< sulfamethoxazole, naproxen< diclofenac, trimethoprim< metoprolol, clarithromycin< climbazole< fluconazole, carbamazepine (**Table 4**). Carbamazepine and fluconazole had a relatively strong sorption capacity to soil, showing the highest *K*_d_ values compared to other compounds, which were less or weakly absorbed by soil. *K*_d_ values were almost similar in the different irrigation treatments, except for metoprolol showing increased *K*_d_ values in 3× TWW.

Since no PhACs were detected in the soil–plant system irrigated with FW, UF values were not calculated for this irrigation treatment. Most of the PhACs did not accumulate in the aerial part of plants in both irrigation treatments and at both T1 and T2, as shown by the absence or very low UF values for stem, shoots, leaves, pulp, and kernel. In contrast, carbamazepine and fluconazole were the only PhACs that translocated to all plant organs as highlighted by their UF values (**Table 4**).

**Figure 4:**
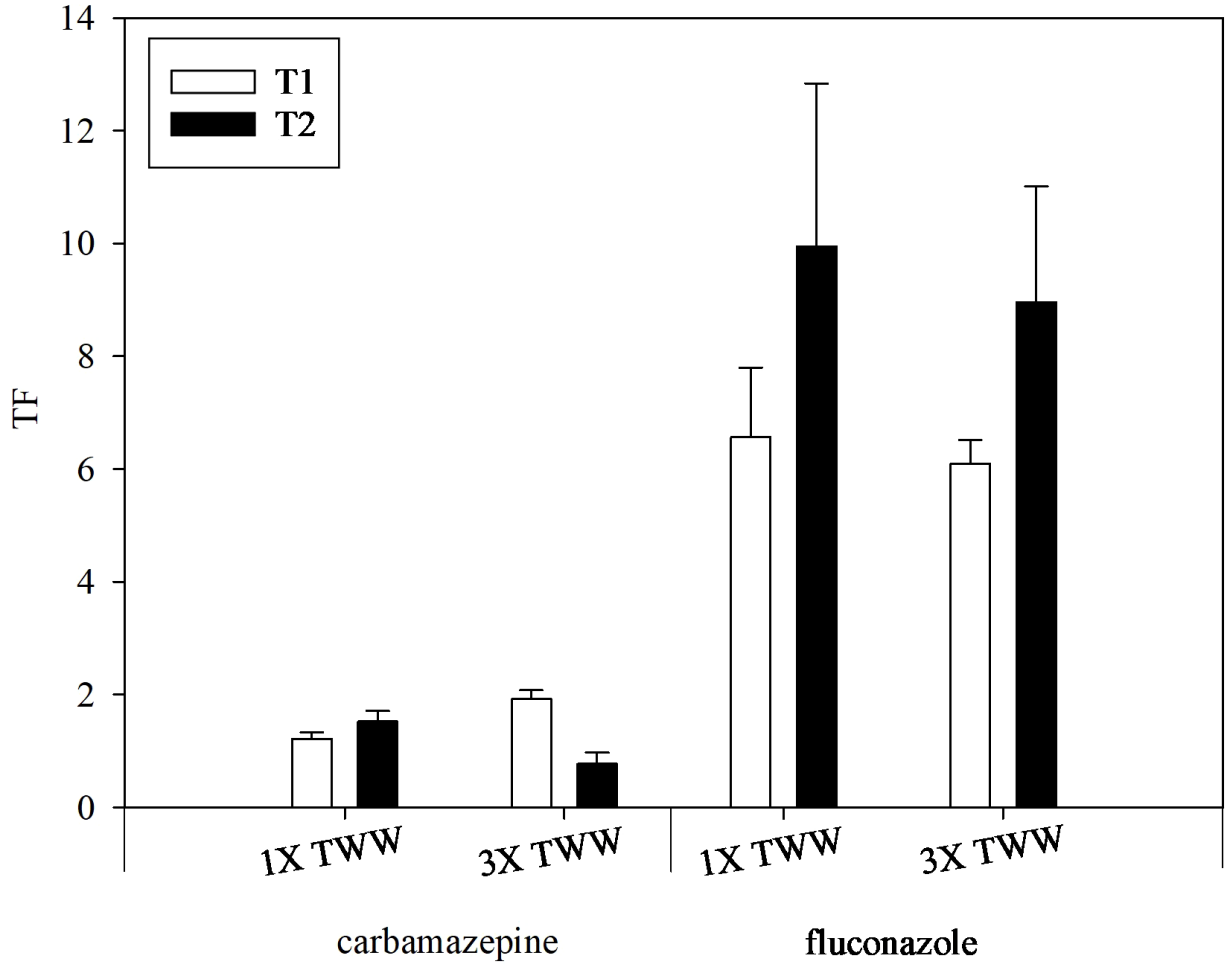
Translocation factor (TF) calculated for two PhACs, carbamazepine and fluconazole, after irrigation with 1× TWW and 3× TWW treatment at T1 and T2. Data are expressed as the mean value and bars are standard errors (*n* = 3).

The greatest UF values for the olive roots were reported for ketoprofen at T1 in both irrigation treatments and for diclofenac and ketoprofen at T2 in the 1× TWW and 3× TWW irrigation treatments, respectively (**Table 4**). In comparison, relatively small UF values for the olive roots were found for the other PhACs, ranging from 0.08 to 0.63 g g^−1^ at T1 and 0.03 to 0.45 g g^−1^ at T2 in both irrigation treatments. Higher UF values were observed in roots for diclofenac in both irrigation treatments at T2 compared to T1 and for metoprolol in the 3× TWW at T1 compared to T2. As reported in **Table 4**, carbamazepine and fluconazole were the only compounds in which UF values were computed for the leaves, stem, shoots, pulp, and kernel, except for metoprolol and clarithromycin showing very low values at T2 in the leaves and stem of 3× TWW irrigation treatment, respectively. UF values calculated for each plant organ at T2 decreased or remained quite steady for carbamazepine and fluconazole, respectively, in the 3× TWW compared to 1× TWW irrigation treatment. On the other hand, the TF was calculated to evaluate the movement of a compound from roots to the aerial parts of the plant (i.e., leaves). TF >1 indicates that a compound is more prone to translocation than to accumulation in roots (Fernandes et al., 2024). The TF was mostly >1 for both compounds, with the only exception of carbamazepine at T2 in the 3× TWW irrigation treatment, which implies a limitation to carbamazepine movement toward leaves in the highest concentration irrigation treatment (**Figure 4**). Since TF is expressed as the ratio between PhACs concentration in leaves to that of roots, both compounds showed a higher concentration in leaves compared to roots, indicating a good root-to-leaf PhAC translocation.

**Figure 5:**
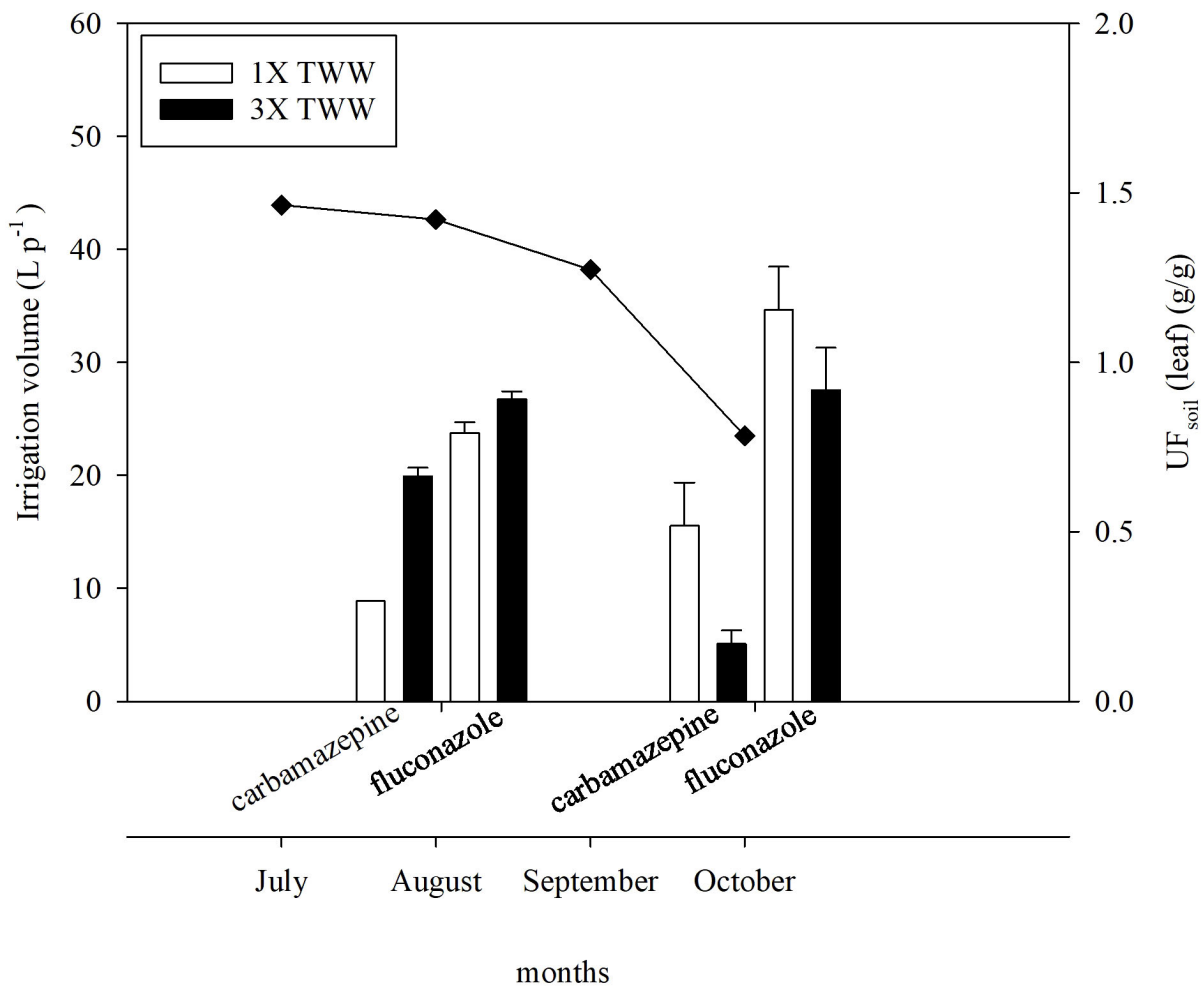
UF calculated considering the concentration in leaf of carbamazepine and fluconazole and irrigation volumes (L) applied each month per plant during the experimental trial.

Considering the importance of irrigation volumes, administered according to the crop and related growing period, in affecting the potential uptake of PhACs, monthly irrigation volumes and changes in the UF calculated for the leaves in carbamazepine and fluconazole among the two different sampling times are shown in **Figure 5**. Irrigation volumes were higher during July and August due to the more demanding environmental conditions and decreased in the following months (September and October). UF values showed an increasing trend at T2 in the 1× TWW for both carbamazepine and fluconazole, whereas they even decreased or remained almost the same at T2 in the 3× TWW for carbamazepine and fluconazole, respectively.

### 3.3 Plant organ biomass assessment

The effect of PhACs on plant growth was also evaluated considering the plant organ biomass accumulation. The plant organ biomass partitioning, expressed as fresh weight (g) and dry matter (%), is shown in **Table 5**.

**Table 5:** Plant organ biomass partitioning, expressed as fresh weight (g) and dry matter (%) of the olive trees irrigated with the three treatments: FW, 1× TWW, and 3× TWW (*n* = 3 ± SE).

Plant organs	Fresh weight (g)			Dry matter (%)		
	FW	1× TWW	3× TWW	FW	1× TWW	3× TWW
Roots (1–5 mm)	171.5 ± 30.2	120.4 ± 10.2	146.8 ± 28.3	33.9 ± 1.5	30.6 ± 1.7	36.7 ± 9.7
Roots (>5 mm)	77.8 ± 18.8	105.0 ± 31.0	102.4 ± 46.1	46.0 ± 4.8	38.8 ± 2.5	44.9 ± 2.6
Stump	478.4 ± 74.6	445.2 ± 45.5	549.6 ± 102.8	59.4 ± 3.1	56.2 ± 0.7	56.5 ± 10.4
Stem	488.7 ± 56.7	516.9 ± 97.2	565.0 ± 56.4	57.1 ± 0.1	56.9 ± 1.7	57.8 ± 1.9
Shoots >2 years	176.3 ± 14.0	193.4 ± 33.4	206.8 ± 10.4	53.4 ± 1.7	56.9 ± 1.4	55.5 ± 0.3
Shoots 2 years	160.4 ± 30.2	101.0 ± 19.7	194.6 ± 8.6	51.9 ± 0.3	57.3 ± 2.9	52.6 ± 0.3
Shoots 1 year	129.4 ± 1.5	126.3 ± 20.1	165.1 ± 7.8	52.3 ± 2.4	52.3 ± 1.6	51.7 ± 2.8
Leaves	284.7 ± 3.2	277.8 ± 44.2	363.1 ± 17.1	47.4 ± 0.6	47.7 ± 1.0	48.2 ± 0.7
Pulp	813.6 ± 8.4	818.7 ± 89.0	864.6 ± 73.4	40.5 ± 1.2	36.7 ± 0.8	37.1 ± 0.5
Kernel	149.6 ± 1.5	150.6 ± 16.4	159.0 ± 13.5	72.5 ± 1.2	77.0 ± 0.4	75.0 ± 1.5
**Total**	2,928.5 ± 119.9	2,855.3 ± 192.2	3,316.8 ± 162.9	512.9 ± 8.5	510.6 ± 4.4	516.0 ± 10.4

No significant differences exist between treatments according to ANOVA (*p* ≤ 0.05) (average data ± standard error).

## 4 Discussion

The present study, focusing on the uptake, translocation, and accumulation of different PhACs applied as a mixture in olive trees, revealed that some PhACs could enter the plants and distribute among aboveground organs, while others remained confined into the soil and roots, showing different distribution dynamics within the soil–plant system. PhACs were applied in mixture to simulate the complex whole of compounds that could actually be found in TWW and enter agricultural environments.

### 4.1 Accumulation of PhACs in soil and roots

Almost all the studied PhACs were mainly detected in the soil and root system at both exposure times and irrigation treatments (**Figures 2**, **3**). In particular, PhACs detected at higher concentrations in the soil showed the highest concentrations in roots in both irrigation treatments (**Figure 2**), which could be related to an increased availability of PhACs for root uptake. Water and dissolved compounds can enter the root through the epidermis of root tips and hairs, cross the cortex, and reach the vascular tissue, through which PhACs can be transported via xylem/phloem to the aerial part of the plant (Miller et al., 2016). The Casparian strip, composed of lignin and suberin, acts as a hydrophobic barrier blocking the apoplastic transport of compounds in the vascular tissue and only those compounds able to cross the lipid bilayer enter the xylem or phloem (Miller et al., 2016; Shi et al., 2022). Compounds that are unable to reach the vascular tissue remain confined in the roots and are not translocated to the aboveground tissues. Uptake and accumulation of PhACs is also affected by their physicochemical properties, including hydrophobicity, chemical structure, and charge, which determine their existence in neutral or ionic forms in the rhizosphere (Christou et al., 2019b).

***Anionic compounds:*** Sulfamethoxazole, ketoprofen, and naproxen, which show similar molecular weight and ionic charge (negative), frequently occurred below their LOQ (**Figure 2**), in agreement with the lower permeability of cell membranes to ionic organic compounds in general (Goldstein et al., 2014). These compounds were found in the plant material of vegetable crops (Goldstein et al., 2014) and root crops (Malchi et al., 2014) but at concentrations much lower than those of nonionic neutral compounds, crossing membranes at a slower rate than non-ionic compounds, reducing accumulation in roots, and thus lowering the uptake compared to neutral compounds, explained with repulsion mechanisms by the negatively charged cell walls and ion trap effects that affect their accumulation in roots (Goldstein et al., 2014). Results showed that they have not been found in plant organs of olive trees other than roots, with higher concentrations in the 3× TWW irrigation treatment (**Figures 2B, D**), probably due to interaction mechanisms that blocked them before reaching the root vascular tissue and avoided their translocation to the aerial part. Sulfamethoxazole, ketoprofen, and naproxen are also characterized by weak interactions and sorption in soil, which is indicated by low soil–water sorption coefficients (*K*_d_ = 0.02–0.16 mL g^−1^) (**Table 4**), in agreement with Zhang et al. (2014). Furthermore, they are characterized by high water solubility and anionic form when soil pH > 7, determining electrostatic repulsion between these compounds and the negatively charged soil particles (De Mastro et al., 2023). Weak acid PhACs were reported to be readily degradable in agricultural soils (Grossberger et al., 2014). The reduced concentration of sulfamethoxazole achieved at T2 in the soil of 1× TWW agrees with what was supported by Zhang et al. (2017), who stated that the lower octanol-water partitioning coefficient (*K*_ow_) and sorption capacity of sulfamethoxazole may lead to its high mobility and removal, hypothesizing a rapid degradation of sulfonamide antibiotics in soil. The two non-steroidal anti-inflammatories—ketoprofen and naproxen—were not detected at all or at low concentrations in soils of both 1× and 3× TWW at both times, partially in agreement with De Mastro et al. (2023), who did not detect these compounds in any of the soil investigated in an experiment conducted on artichoke, which could be probably related to the fewer irrigation interventions carried out compared to olive plants, whereas sulfamethoxazole has always been detected above the LOQ in soils of both 1× and 3× TWW at both times but at rather low concentrations compared to other PhACs, in agreement with De Mastro et al. (2023), who reported lower concentrations of sulfamethoxazole in soils, probably attributable to the same reason given for previous compounds. Diclofenac is a hydrophobic (log*K*_ow_ > 4) and negatively charged ionic (p*K*_a_ = 4.1) compound in the soil environment (with a pH of 7.7). This PhAC was found at intermediate concentrations in soil and roots of 1× and 3× TWW at both T1 and T2 compared to other PhACs (**Figure 2**), whereas it was not detected in the aerial parts of olive plants (**Table 4**). This result is supported by other studies corroborating that highly hydrophobic compounds, such as diclofenac, could be strongly bound to root surfaces, thus limiting their translocation into the plant (Schnoor et al., 1995). Highest concentrations of diclofenac were found in the roots of three lettuce varieties hydroponically cultivated compared to leaves, suggesting that this compound underwent a very slow translocation process (González García et al., 2018). However, other studies found diclofenac in tomato samples, even fruits, under long-period wastewater irrigation (Christou et al., 2017; Bueno et al., 2022), explaining the accumulation of ionic compounds in plant organs due to different pH levels (Picó et al., 2019).

***Cationic compounds:*** Metoprolol, with a comparable molecular weight but a positive ionic charge, was found at significantly higher concentrations in olive roots compared to the negatively charged PhACs (**Figures 2B, D**) and at a very low concentration in leaves of the 3× TWW irrigation treatment (**Table 4**), in partial agreement with results obtained in cucumber where positively charged metoprolol was detected at higher concentrations (Goldstein et al., 2014). Moreover, relatively high concentrations were found in soils that were maintained over time (**Figures 2A, C**), according to previous studies, suggesting a recalcitrant behavior and accumulation capacity in soils of metoprolol (Grossberger et al., 2014). Trimethoprim, falling under the weak to moderate base (p*K*_a_ ≥ 6), can be found in its neutral and cationic forms in the rhizosphere (Christou et al., 2016). Chemical species of trimethoprim existing in soil, depending on the acid–base coefficient (p*K*_a_) and soil solution pH (Shi et al., 2022), explained its relatively high concentrations found in roots, since positively charged trimethoprim could be absorbed by the negatively charged root surface, and in leaves, since it could also be taken up as a neutral compound and reach the aboveground tissues following the transpiration flow, in leafy and fruit vegetables (Herklotz et al., 2010; Tanoue et al., 2012). In olive plants, trimethoprim was detected only in roots (**Figure 2**; **Table 4**), partially in agreement with studies carried out on vegetables where trimethoprim was found in both roots and leaves but with higher concentrations in roots (Herklotz et al., 2010; Tanoue et al., 2012). Both metoprolol and trimethoprim were found at relatively high and similar concentrations in the soil of 1× TWW (**Figure 2A**), whereas trimethoprim concentration in soil was significantly reduced compared to metoprolol in the soil of 3× TWW (**Figure 2C**). Sorption of PhACs on soil particles, depending on particular soil properties, is a main parameter affecting PhACs mobility within the porous media and consequently root uptake availability (Kodešová et al., 2015). These PhACs have similar physicochemical properties (**Supplementary Table S1**), and their high accumulation in soil can be ascribed to sorption processes of cation forms in the soil, since sorption coefficients for both trimethoprim and metoprolol were positively correlated and thus mostly dependent on the base cation saturation and cation exchange capacity (Kodešová et al., 2015). Clarithromycin and climbazole also showed high concentrations in soils of both 1× and 3× TWW (**Figures 2A, C**). Clarithromycin differs from other PhACs because of its higher molecular weight (**Supplementary Table S1**), which could be probably one of the causes of its high sorption in soil, since various groups may be involved in sorption (Kodešová et al., 2015), thus explaining the high concentration found in the soil. Furthermore, clarithromycin was not translocated from roots to aerial parts of olive plants (**Table 4**) probably due to its high molecular volume and weight, which are factors affecting compound translocation, since smaller molecules were found to easily cross the Casparian strip in the root (Miller et al., 2016; Shi et al., 2022). Climbazole, reported as a weak base with a p*K*_a_ of 6.49, coexists in both its neutral and cationic states at neutral or high pH, whereas at low soil pH (acidic), the positively charged form, i.e., the protonated climbazole fraction, prevails (Richter et al., 2013; Manasfi et al., 2021). Presumably, considering soil, irrigation water, and climbazole physicochemical properties (**Tables 1**, **2**; **Supplementary Table S1**), it was more present in the soil in its neutral molecule. The neutral compound of climbazole with a log*K*_ow_ of 3.33 can be classified as hydrophobic and therefore could strongly interact with the organic portion of the soil (Richter et al., 2013), increasing the adsorption in soil and explaining the high concentration found also in soil (**Figures 2A, C**). In particular, clarithromycin, metoprolol, and climbazole concentrations in roots were higher than other PhACs because their larger accumulation in soil probably increased the available concentration for plant uptake (**Figure 2**), although these compounds did not translocate and accumulate in leaves of olive plants but remained confined into roots (**Table 4**), differently from results obtained in lettuce crop (Manasfi et al., 2021). Fluconazole, among azole compounds, is a base that can be dissociated and found both in the neutral and ionized species depending on pH conditions and a more hydrophilic compound (log*K*_ow_ = 0.25) compared to other PhACs (García-Valcárcel et al., 2016). Considering soil and irrigation water pH values (**Tables 1**, **2**), fluconazole is presumably primarily in the non-ionized neutral form, which can more efficiently cross the root membrane by diffusion compared to ionized forms that can be accumulated by the cell, which is negatively charged on the membrane (García-Valcárcel et al., 2016). The high concentration of fluconazole detected in soils (**Figures 3B, D**), although higher than that measured in other studies carried out on vegetable crops (De Mastro et al., 2023; Denora et al., 2023), confirmed the high persistence of this compound, whose concentrations tended to increase in soils irrigated with differently spiked TWW over time.

***Neutral compounds:*** Since carbamazepine is mainly present in the environment in its neutral form, with a relatively low log*K*_ow_ (**Supplementary Table S1**), it is not expected to be extensively sorbed in soil; however, other factors can affect the sorption behavior of the compound (Calisto and Esteves, 2012). Important sorbents for non-polar and moderately polar neutral organic compounds in the bulk soil, such as SOM, control and affect the PhACs’ available concentration and therefore the fraction effectively available for root uptake (Miller et al., 2016). Carbamazepine is an intermediate hydrophobic, weakly acid (2 ≤ p*K*_a_ ≤ 6) and non-polar compound whose sorption in soil is mainly controlled by the organic carbon content (Zhang et al., 2010; Kodešová et al., 2015). The high concentration of carbamazepine detected in soil (**Figures 3A, C**) could be explained by both its high persistence, promoting its accumulation over time significantly in 3× TWW, and the relatively higher content of organic carbon (**Table 1**) compared to other studies (De Mastro et al., 2023; Denora et al., 2023), acting on the soil sorption of the carbamazepine.

The highest concentrations of climbazole, carbamazepine, and clarithromycin were also measured in soil samples in experiments carried out on lettuce, which were well correlated with *K*_d_ values, accounting for their retention in soil (Manasfi et al., 2021), and tomato (Denora et al., 2023). Since the mobility of PhACs increases with decreasing sorption coefficients, affecting their potential transport within soil and uptake by plants, other properties, such as half-lives, persistence, and degradation, have to be considered. Previous studies suggested that carbamazepine can be considered as a persistent organic contaminant when it is introduced to soils (Grossberger et al., 2014; Pacholak et al., 2022) and reported the high persistence of carbamazepine combined with its weak sorption in soil, allowing a large fraction of this PhAC to remain in soil, which is readily available to root uptake, leading to relatively high accumulation in plants and causing adverse effects on both environmental and human health (Koba et al., 2016; Li et al., 2019).

### 4.2 Translocation of PhACs: analysis of carbamazepine and fluconazole

In olive plants, fluconazole and carbamazepine showed a high transport toward leaves, as also expressed by the TF value (**Figure 4**), which is in line with results obtained by Sochacki et al. (2021). Perhaps, the translocation of a compound in aerial plant tissue is more dependent on its lipophilicity, indicated by the log*K*_ow_ value (Christou et al., 2019b). In particular, the translocation of hydrophilic PhACs, such as fluconazole, is promoted through the Casparian strip, acting as a hydrophobic barrier, compared to more hydrophobic compounds (Shi et al., 2022). The fact that both carbamazepine and fluconazole were detected in leaves of olive plants (**Figure 3**; **Table 4**) demonstrated that these compounds can be transported from root to leaf, according to previous studies carried out on vegetable crops, such as tomato, cucumber, artichoke, and lettuce (Goldstein et al., 2014; García-Valcárcel et al., 2016; De Mastro et al., 2023; Denora et al., 2023). Generally, small-sized and neutrally charged compounds, such as carbamazepine, can effectively be taken up by plants, moving along the root with water flow and into the root following the symplastic pathway, easily crossing the Casparian strip and entering the xylem. These compounds can be transported to the aerial part of the plant following the transpiration stream (passive process), accumulating mainly in the transpiring organs, such as leaves (Goldstein et al., 2014; Malchi et al., 2014; Riemenschneider et al., 2017; Ben Mordechay et al., 2018; Chuang et al., 2019; Kodešová et al., 2019). The bioaccumulation of carbamazepine has been found to be higher in leaves than in the stem and roots in hydroponic cultures of both cucumber and tomato plants (Shenker et al., 2011; Riemenschneider et al., 2017). On the other hand, the concentration of carbamazepine detected in leaves of olive plants did not statistically differ from that found in roots at both T1 and T2 in 1× TWW and at T2 in 3× TWW, showing an increase compared to the concentration in roots only at T1 in 3× TWW (**Figures 3A, C**). Instead, concentration in leaves was always significantly different from the concentration detected in stem and shoots (**Figures 3A, C**). Furthermore, a plateau was observed in the concentration of carbamazepine in leaves, maintaining almost steady values, compared to that of fluconazole, which showed significant increases over time in both irrigation treatments (**Figures 3B, D**). To evaluate the transport of both carbamazepine and fluconazole from root to leaves, the TFs were determined (**Figure 4**). In particular, both carbamazepine and fluconazole showed TF > 1, which indicates that compounds are more prone to move from the roots toward leaves and fluconazole was characterized by the highest TF values (6–10) compared to carbamazepine (0.8–2) (**Figure 4**). After root absorption, these PhACs are transported upward in the aerial part of plants through the xylem following the water flow driven by the water potential gradient occurring during transpiration, according to the evapotranspiration demand (García-Valcárcel et al., 2016; Wei et al., 2023). Differences found in the TF of these PhACs can be ascribed to different log*K*_ow_ values, equal to 0.25 and 2.45 for fluconazole and carbamazepine respectively, since more hydrophilic compounds are translocated to a major extent compared to more hydrophobic compounds (García-Valcárcel et al., 2016). The ability of olive plants to uptake and accumulate PhACs from spiked TWW irrigated soil in the belowground (i.e., roots) and aboveground plant tissues (i.e., stem, shoots, leaves, and fruits) was also assessed through the determination of UF, which was established as the ratio of each compound in a specific plant organ and in soil (**Table 4**). Patterns analyzed suggested that all compounds that reached with greater difficulty the aerial part of vegetable crops and tended to preferentially accumulate in the roots have not been detected at all in the aerial tissues of olive trees, such as stem, shoots, leaves, and fruits, and they corresponded to ionic and charged compounds or with higher log*K*_ow_ values, which probably interacted at different levels. Higher UF values (computed for the roots) can be indicative of compounds mainly adsorbed to roots and in some cases taken up by roots (**Table 4**), e.g., cationic states or high lipophilicity of the compound, the latter responsible for concentration on roots by partitioning into lipophilic components (García-Valcárcel et al., 2016), and thus less able to move upward. Carbamazepine and fluconazole showed higher UF values (computed for the leaves), indicating an effective accumulation in the main transpiring organs of the plant, and very low UF values for the roots (**Table 4**), which could be indicative of PhACs absorption process by the roots, as previously reported for fluconazole (García-Valcárcel et al., 2016). Carbamazepine and fluconazole were also detected in the edible parts of olive plants in both irrigation treatments (**Figure 3**). Previous studies found several PhACs, such as fluconazole, carbamazepine, metoprolol, clarithromycin, climbazole, and sulfamethoxazole, in tomato fruits, with the highest concentrations detected for carbamazepine and fluconazole, respectively (Denora et al., 2023). A lower number of PhACs was also reported to accumulate in several fruits, such as citrus, tomato, banana, and avocado (Ben Mordechay et al., 2022). Fruits exhibited the lowest number of PhACs detected compared to leafy greens (18), particularly avocado (10), tangerine (9), orange (8), tomato (7), and banana (6) (Ben Mordechay et al., 2021). In the present study, PhACs detected in edible parts of olive plants were only 2 (fluconazole and carbamazepine) of the total 10 investigated (**Figure 3**).

Neutral forms of PhACs exhibited a greater uptake by olive plants compared to ionic species, and, more in detail, cationic species showed higher bioaccumulation than anionic forms, confirming previous results reported in vegetable crops (Goldstein et al., 2014; Li et al., 2019; Manasfi et al., 2021). This is probably due to different plant physiology and *in planta* processes between perennial fruit trees and their sink organs and annual vegetables.

### 4.3 Factors affecting PhACs uptake and accumulation

The amount of PhACs distributed through the 3× TWW was significantly higher than those usually found in municipal wastewater, simulating extreme conditions with the aim of investigating the PhACs behavior and mechanisms involved in plant uptake and translocation.

Overall results showed that PhACs concentrations in soil and plant samples were found to be higher in the irrigation treatment fortified with the highest dose of the PhACs mixture, i.e., 3× TWW, according to Wu et al. (2014), highlighting the fact that the concentration of PhACs in the irrigation water is a key factor in the uptake process (Christou et al., 2019b). Another important factor that should be considered when discussing the potential of crop plants to uptake PhACs concerns crop plants’ irrigation requirement and irrigation period. Irrigation requirement values are considered to affect the ability and the potential for uptake of PhACs by plants, since higher irrigation requirement values have been found to correspond to a greater ability to uptake and accumulate PhACs, by the xylem stream (Christou et al., 2019b). Differential water consumption, influenced by environmental conditions and other crop-specific factors, such as growing period and irrigation regime, may produce PhACs accumulation discrepancies in plants (Goldstein et al., 2014; Pullagurala et al., 2018). Reduced irrigation volume distributed in the second part of the irrigation season (September, October, 2021) (**Figure 5**), driven by lower environmental evapotranspiration demand, may have affected the potential uptake and accumulation of specific PhACs, as shown by decreasing concentrations of some PhACs in soil and roots at T2 (**Figure 2**), in addition to possible degradation processes involved in secondary metabolism. In particular, some PhACs, identified as more susceptible to degradation, such as ketoprofen, sulfamethoxazole, and naproxen, were more easily removed from the soil–plant system when the irrigation volume decreased, whereas those compounds identified as more persistent, such as carbamazepine and fluconazole, tended to maintain an almost constant and increased concentration in the leaves over time, respectively (**Figure 5**). This is in agreement with Ben Mordechay et al. (2022) and Bagheri et al. (2021) in which a plateau in carbamazepine leaf accumulation was reported, probably resulting from a steady-state condition achieved when uptake equals *in planta* decomposition. Furthermore, results that showed that carbamazepine continued to increase in the root but remained constant in the leaves in the highest dose irrigation treatment (3× TWW) (**Figure 3**) can suggest the involvement of mechanisms in the roots blocking the transport within plant root cells. The plant organ biomass was very similar among the three treatments, suggesting no phytotoxic effects for the 10 PhACs. No statistically significant differences in fresh weight and dry matter (%) were found among plant organs irrigated with FW, 1× TWW, and 3× TWW treatments (**Table 5**). These results are in agreement with previous studies that reported the absence of effects on plant growth cultivated with soils containing pharmaceuticals deriving from biosolids or TWW for irrigation (Wu et al., 2013; Beltrán et al., 2020).

## 5 Conclusions

Crop irrigation carried out with TWW represents a recognized opportunity to address the challenges of increasing water scarcity. However, attention should be paid to the potential exposure of humans to organic contaminants, with possible implications for both human and environmental health. This study investigated the capacity of olive plants grown in pots to uptake and translocate PhACs deriving from spiked TWW used for irrigation. Specifically, the uptake and distribution patterns of 10 PhACs were evaluated in the irrigation water–soil–plant system along one irrigation season. Findings showed that most PhACs were able to accumulate in soils and proportionally exclusively in the belowground part of the plant, i.e., the roots, related to the physicochemical properties of each compound. Among these PhACs, moderate hydrophobic carbamazepine and high hydrophilic fluconazole were the only compounds able to move from the roots and translocate to the aboveground organs, including the edible ones. Therefore, on one hand, particular attention should be paid to these compounds that showed a higher tendency to translocate to the edible part, due to their physicochemical properties; on the other hand, those compounds with higher degradation and lower uptake need further investigations for the occurrence of secondary metabolites. Results indicate that due to prolonged TWW irrigation, the soil and different plant compartments can act as a sink for PhACs throughout the woody plant life cycle, underlying the need for future research for a long-term evaluation of distribution of PhACs in the soil–plant system to assess potential effects on human and environmental health. Considering the importance of the soil acting as a medium capable of adsorbing and retaining these compounds and of irrigation water as a carrier for their uptake and translocation into plants, innovative soil and irrigation strategies should be developed to reduce the uptake by plants and the accumulation in edible organs, allowing a safer use of TWW for crop irrigation.

## Data availability statement

The raw data supporting the conclusions of this article will be made available by the authors, without undue reservation.

## Author contributions

AM: Conceptualization, Data curation, Formal analysis, Investigation, Methodology, Writing – original draft, Writing – review & editing, Resources. AP: Writing – original draft, Data curation, Formal analysis, Investigation, Methodology, Resources, Writing – review & editing. MC: Data curation, Formal analysis, Investigation, Methodology, Writing – original draft, Resources, Writing – review & editing. RDB: Methodology, Writing – original draft. GB: Resources, Writing – review & editing. FDM: Resources, Writing – review & editing. SM: Resources, Writing – review & editing. CDC: Resources, Writing – review & editing. CS: Resources, Writing – review & editing. BD: Conceptualization, Funding acquisition, Methodology, Supervision, Writing – review & editing, Investigation, Resources.
