# Supplementary material for: Uptake and translocation of pharmaceutically active compounds by olive tree (Olea europaea L.) irrigated with treated municipal wastewater
Source: Front Plant Sci. 2024 May 2;15:1382595. doi: 10.3389/fpls.2024.1382595 (PMC11096453; doi:10.3389/fpls.2024.1382595)
Supplement: Supplementary file 1 [file DataSheet_1.docx]

***Supplementary Material***

**Uptake and translocation of Pharmaceutically Active Compounds by olive tree (*Olea europaea* L.) irrigated with treated municipal wastewater**

**Alba N. Mininni^1*^, Angela Pietrafesa^1^, Maria Calabritto^1^, Roberto Di Biase^1^, Gennaro Brunetti^2^, Francesco De Mastro^2^, Sapia Murgolo^3^, Cristina De Ceglie^3^, Carlo Salerno^3^, Bartolomeo Dichio^1^**

^1^ University of Basilicata, Department DICEM, Via Lanera 20, 75100, Matera, Italy

^2^ University of Bari, Department of Soil, Plant, and Food Science, Piazza Umberto I, 70121, Bari, Italy

^3^ CNR, Istituto di Ricerca Sulle Acque, Department of Bari, V.le F. De Blasio 5, 70132, Bari, Italy

*** Correspondence:**Alba N. Mininni
alba.mininni@unibas.it

# Supplementary Figures and Tables

## Supplementary Figures


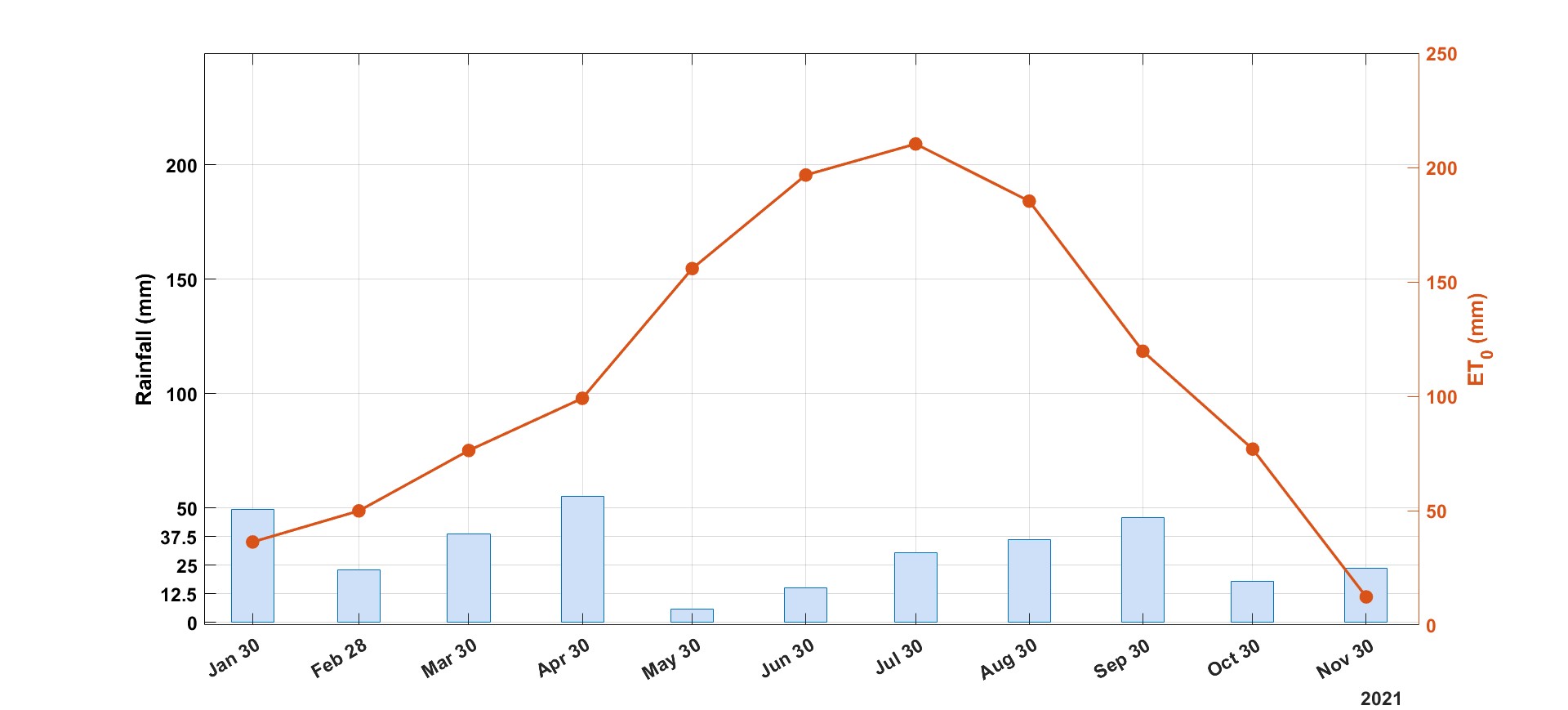


**FIGURE S1** Monthly reference evapotranspiration (ET_0_) and rainfall for 2021 obtained from the AASD Pantanello weather station of ALSIA Research Institute.

- 1. **Supplementary Tables**

**TABLE S1** Physico-chemical properties of the Pharmaceutically Active Compounds (PhACs) selected in this study.

| **PhACs** | **Molecular weight (g mol^-1^)** | **Chemical Structure** | **Therapeutic class** | **Water**  **Solubility**  **mg L^-1^** | **logK_OW_** | **pKa** |
| --- | --- | --- | --- | --- | --- | --- |
| Clarithromycin | 748.0 | 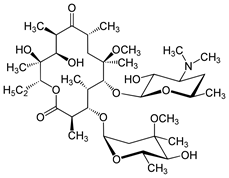 | antibiotic | 1.69 at 25°C | 3.16 | 8.99 |
| Sulfamethoxazole | 253.3 | 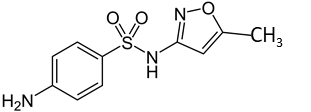 | antibiotic | 610 at 37°C | 0.89 | 1.6 |
| Trimethoprim | 290.3 | 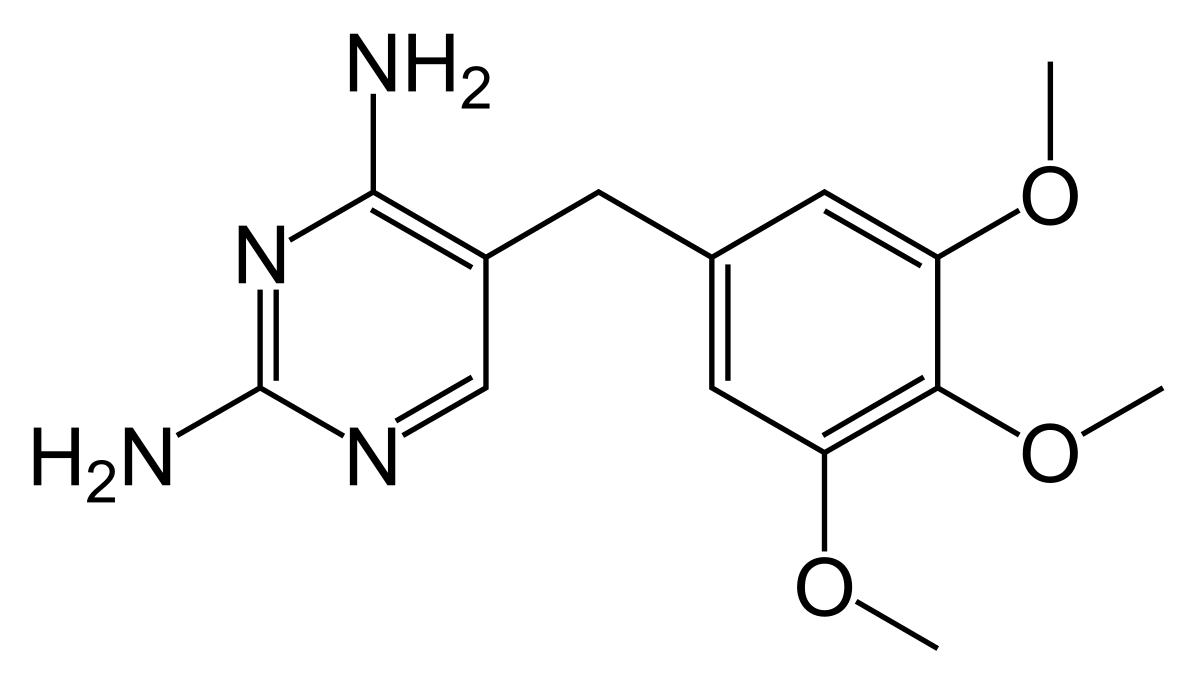 | antibiotic | 400 at 25°C | 0.91 | 7.12 |
| Ketoprofen | 254.3 | 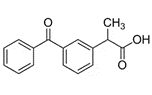 | anti-inflammatory | 51 at 22°C | 3.12 | 4.45 |
| Carbamazepine | 236.27 | 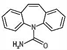 | antiepileptic | 18 at 25°C | 2.45 | 13.9 |
| Diclofenac | 296.1 | 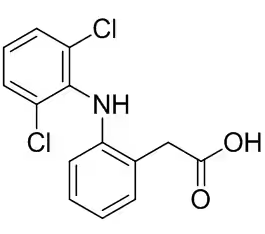 | anti-inflammatory | 2.37 at 25°C | 4.51 | 4.15 |
| Metoprolol | 267.36 | 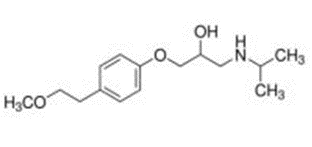 | beta-blocker | 0.4 at 25°C | 1.88 | 9.7 |
| Fluconazole | 306.27 | 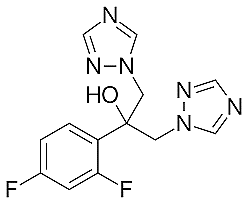 | antifungal | 4.36 at 25°C | 0.25 | 2.27 |
| Climbazole | 292.76 | 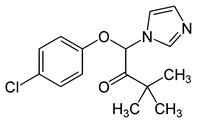 | antifungal | 50 at 25°C | 3.76 | 7.5 |
| Naproxen | 230.26 | 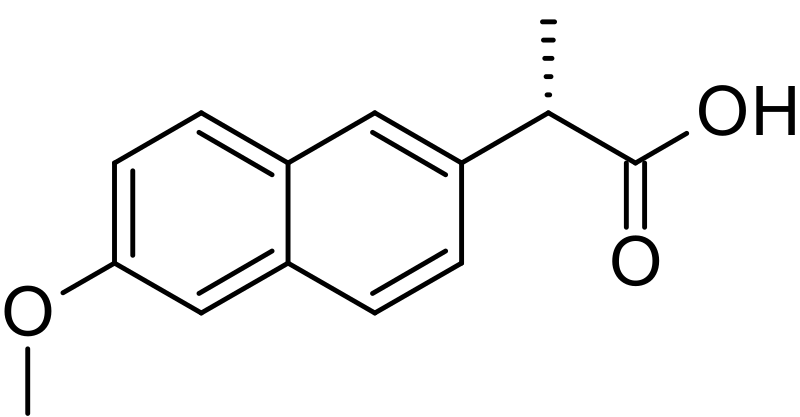 | anti-inflammatory | 15.9 at 25°C | 3.18 | 4.15 |
